# Disposable Electrochemical Sensors for Highly Sensitive Detection of Chlorpromazine in Human Whole Blood Based on the Silica Nanochannel Array Modified Screen-Printed Carbon Electrode

**DOI:** 10.3390/molecules27238200

**Published:** 2022-11-24

**Authors:** Qianqian Han, Tongtong Zhang, Meifang Wang, Fei Yan, Jiyang Liu

**Affiliations:** 1 Key Laboratory of Surface & Interface Science of Polymer Materials of Zhejiang Province, Department of Chemistry, Zhejiang Sci-Tech University, Hangzhou 310018, China; 2 Key Laboratory of Integrated Oncology and Intelligent Medicine of Zhejiang Province, Department of Hepatobiliary and Pancreatic Surgery, Affiliated Hangzhou First People’s Hospital, Zhejiang University School of Medicine, Hangzhou 310006, China

**Keywords:** vertically ordered mesoporous silica films, screen-printed carbon electrodes, electrochemically reduced graphene oxide, chlorpromazine, electrochemical sensors, human whole blood

## Abstract

Rapid and highly sensitive quantitative analysis of chlorpromazine (CPZ) in human whole blood is of great importance for human health. Herein, we utilize the screen-printed carbon electrodes (SPCE) as the electrode substrates for growth of highly electroactive and antifouling nanocomposite materials consisting of vertically ordered mesoporous silica films (VMSF) and electrochemically reduced graphene oxide (ErGO) nanosheets. The preparation of such VMSF/ErGO/SPCE could be performed by using an electrochemical method in a few seconds and the operation is controllable. Inner ErGO layer converted from graphene oxide (GO) in the growth process of VMSF provides oxygen-containing groups and two-dimensional π-conjugated planar structure for stable fabrication of outer VMSF layer. Owing to the π-π enrichment and excellent electrocatalytic abilities of ErGO, electrostatic preconcentration and antifouling capacities of VMSF, and inherent disposable and miniaturized properties of SPCE, the proposed VMSF/ErGO/SPCE sensor could be applied for quantitative determination of CPZ in human whole blood with high accuracy and sensitivity, good stability, and low sample consumption.

## 1. Introduction

Chlorpromazine (3-(2-chloro-10*H*-phenothiazin-10-yl)-*N*,*N*-dimethyl-propan-1-amine, CPZ), as a class of phenothiazine derivatives with aliphatic side chains, is an important antipsychotic drug, which has been widely used to treat personality disorders and various psychotic disorders, such as adult manic depression or schizophrenia [1,2]. Excessive dose or concentration could pose severe problems to human health, such as abnormal pigmentation of conjunctiva and eyelids, respiratory disorders, cataracts, and dysregulation [3]. Therefore, rapid and highly sensitive quantitative analysis of CPZ is of great importance. To date, numerous techniques have been developed to realize the determination of CPZ, such as high-performance liquid chromatography [4], spectrophotometry [5], capillary electrophoresis [6,7], fluorometry [8], gas chromatography coupled with mass spectrometry [9], and electrochemistry [10,11]. Electrochemical sensors have shown the advantages of rapidity, simplicity, high sensitivity, and inexpensiveness and received considerable attention [12]. Researchers have reported the exploitation of various nanomaterials to modify the electrodes for CPZ detection [2,13]. Chen group developed a peas-like strontium molybdate catalyst by using a sonochemical approach and used this as electrode material for CPZ detection in pharmaceutical and human urine samples [2]. Liu group prepared nitrogen-doped carbon dots/cuprous oxide composite via hydrothermal and wet chemical method and investigated its electrochemical performance for CPZ detection in human urine and pharmaceuticals [13]. However, direct electrochemical analysis of CPZ in rather complex human whole blood samples has never been reported, to the best of our knowledge. This is because electrochemical sensors will suffer from the severe surface fouling issues resulting from the nonspecific absorption of biological macromolecules (e.g., lipids, polysaccharides, cells, and proteins). In this situation, electrochemical sensing interfaces were passivated and electrode performances were significantly declined. Therefore, developing convenient and low-cost electrochemical sensors for sensitive and antifouling determination of CPZ is highly needed.

Many researchers have employed varieties of antifouling materials to minimize or avoid surface fouling of electrodes, such as polymers [14], self-assembled monolayers [15], and porous films [16,17,18,19]. Vertically ordered mesoporous silica films (VMSF), also named as silica nanochannel array, have become attractive antifouling materials for direct electrochemical analysis of ions [20,21], small biological molecules [22,23,24], pesticides [25], explosives [26], environmental pollutants [27,28], drugs [29], and biomarkers [30,31,32,33,34] in complicated real samples. Due to the highly ordered and uniform nanochannels with a diameter of 2~3 nm and high density of silanol groups, VMSF displays a small, negatively charged, and hydrophobic microenvironment, which could effectively prevent the surface fouling through size, electrostatic, and hydrophilic effects [35,36]. Moreover, reversal of electrostatic and hydrophilic properties could be obtained by simply tailoring the silica nanochannels of VMSF, showing wide applications for in vivo and in vitro analysis of practical samples [37,38]. Screen-printed carbon electrodes (SPCE) have emerged as disposable, miniaturized and cheap electrode substrates, which could integrate with VMSF and further demonstrate great potential for on-site detection [39,40].

In this work, we use the electrochemically reduced graphene oxide (ErGO) nanosheets as stable and electroactive layer for preparation of VMSF on the SPCE, which is able to sensitively determine CPZ. Such obtained sensor consisting of an outer VMSF layer and inner ErGO layer, designated as VMSF/ErGO/SPCE, could be controllably prepared by an electrochemical method in a few seconds. Graphene oxide (GO) precoated onto the SPCE was electrochemically converted to the ErGO in the growth process of VMSF, whose oxygen-containing groups and two-dimensional π-conjugated planar structure could favor the stable growth of VMSF on the SPCE and meanwhile, promote the electrocatalytic activity. With the combination of electrostatic enrichment and antifouling abilities of VMSF, π-π preconcentration and excellent electroactive capacities of ErGO, and inherent properties of SPCE, the proposed VMSF/ErGO/SPCE could be applied for direct and disposable detection of CPZ in human whole blood with rather low sample consumption.

## 2. Results and Discussion

### 2.1. Fabrication and Characterization of VMSF/ErGO/SPCE

**Figure 1:**
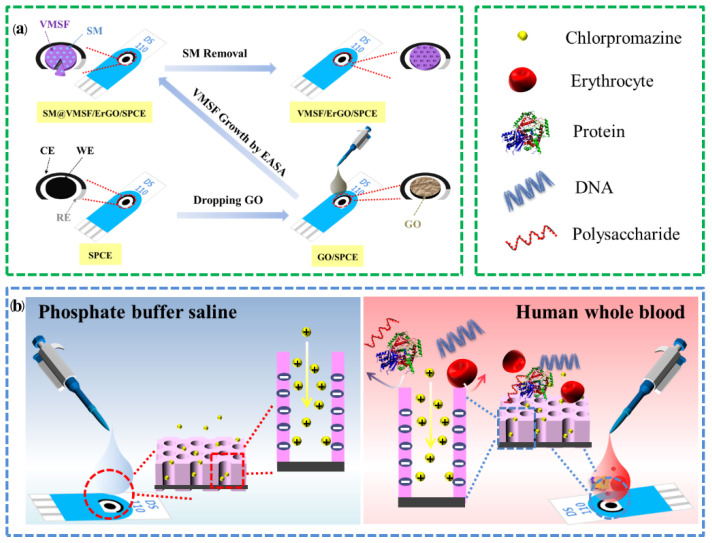
Schematic illustration for the preparation of VMSF/ErGO/SPCE (**a**) and the electrochemical detection of CPZ in PBS and human whole blood samples (**b**).

**Figure 2:**
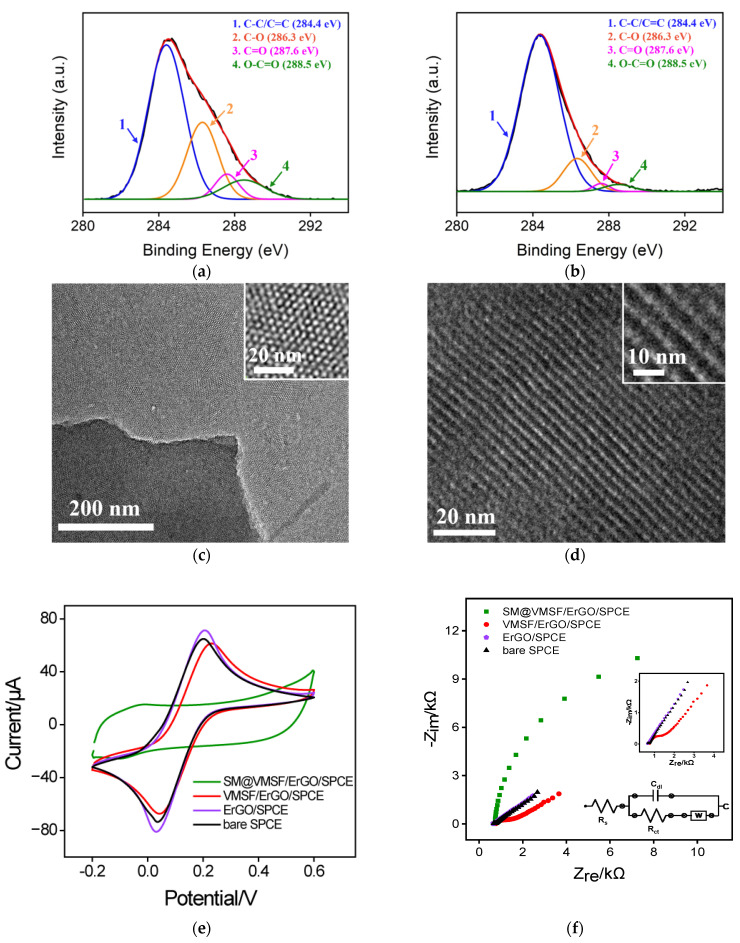
Carbon 1s XPS profiles of GO/SPCE (**a**) and ErGO/SPCE (**b**). Top-view (**c**) and cross-sectional view (**d**) TEM images of VMSF. The insets are the corresponding TEM images with high magnification. CV (**e**) and EIS (**f**) plots of the bare SPCE, ErGO/SPCE, VMSF/ErGO/SPCE, and SM@VMSF/ErGO/SPCE in 0.1 M KCl solution containing 2.5 mM Fe(CN)_6_^3–/4–^. Insets in (**f**) are amplified plots of bare SPCE and ErGO/SPCE, and the fitting equivalent circuit diagram. In addition, *R*_ct_, *R*_s_, *W*, and *C* denote the charge-transfer resistance, uncompensated solution resistance, mass-transport-related Warburg element, and capacitance of ErGO/solution interface, respectively.

Graphene-based materials (e.g., 0D graphene quantum dots [41,42], 2D graphene nanosheets [43], and 3D porous graphene [44]) usually exhibit novel optical/electrical/catalytic properties [45,46,47,48] and have been widely used to construct various sensing systems with improved analytical performance [49,50,51]. Figure 1a illustrates the fabrication process of VMSF/ErGO/SPCE. GO was first dropped onto the SPCE surface to obtain GO-modified SPCE (GO/SPCE). Then, VMSF was prepared onto the GO/SPCE surface by using EASA method, namely a constant electrochemical reduction process, in which the electrochemical reduction in GO simultaneously occurred. Note that the presence of ErGO plays an important role in the stable growth of VMSF. On the one hand, ErGO bearing amounts of negative charge favor the electrostatic adsorption of templated surfactant micelles (SM) and promote the growth of VMSF. On the other hand, ErGO has oxygen-containing groups, which could form O–Si–O chemical bonds between ErGO and VMSF. After extracting the SM from the nanochannels, the resulting electrode was SPCE-modified with hybrid materials consisting of inner ErGO layer and outer VMSF layer, designated as VMSF/ErGO/SPCE. Variation from GO to ErGO was confirmed by XPS. As shown in Figure 2a,b, four characteristic peaks displayed at the 284.4 eV (C–/C=C), 286.3 eV (C–O), 287.5 eV (C=O), and 288.5 eV (O–C=O) were observed at the GO/SPCE. The latter three characteristic peaks corresponding to the oxygen-containing groups decline apparently at the ErGO/SPCE, indicating that GO onto the SPCE has been electrochemically reduced to the ErGO. TEM was used to characterize the morphology of VMSF and the results are shown in Figure 2c,d. As seen from the top-view TEM image (Figure 2c), VMSF processes rather regular and hexagonally aligned nanopores over a large area and the diameter is ca. 2~3 nm. The vertically aligned nanochannels are parallel to each other (Figure 2d). Integrity and electrostatic selectivity of VMSF were proved by electrochemical techniques including cyclic voltammetry (CV) and electrochemical impedance spectroscopy (EIS). As displayed in Figure 2e, in comparison with the bare SPCE, Fe(CN)_6_^3–/4–^ could generate slightly improved redox signals at the ErGO/SPCE, which is due to the good electron transfer capacity of ErGO. The as-prepared SM@VMSF/ErGO/SPCE has SM confined in the nanochannels and is adverse to the access of Fe(CN)_6_^3–/4–^, leading to the capacitive current and suggesting the intact VMSF on the ErGO/SPCE surface. After SM removal, well-defined redox signals of Fe(CN)_6_^3–/4–^ were recovered at the VMSF/ErGO/SPCE and decreased signals were ascribed to the electrostatic effect of VMSF bearing negative charge. The diameter of the semicircle in the high-frequency region displayed in Figure 2f indicates the charge transfer resistance (*R*_ct_) shown in Randles equivalent circuit (inset of Figure 2f). It could be found that variation in *R*_ct_ is consistent with that of voltammetric responses shown in Figure 2e. Note that the *R*_ct_ of VMSF/ErGO/SPCE slightly decreased and was comparable to that of bare SPCE and ErGO/SPCE, demonstrating the proposed VMSF/ErGO/SPCE with open channels favors the mass transport through the silica nanochannels to the underlying electrode. All above results indicate the successful preparation of VMSF/ErGO/SPCE sensor.

### 2.2. Electrochemical Behavior of CPZ at the VMSF/ErGO/SPCE

**Figure 3:**
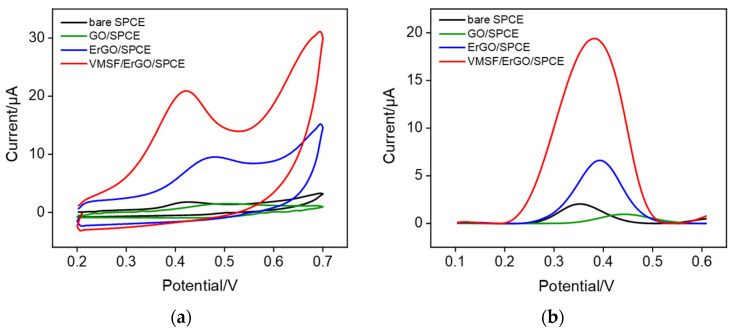
(**a**) CV and (**b**) DPV curves of bare SPCE (black), GO/SPCE (green), ErGO/SPCE (blue), and VMSF/ErGO/SPCE (red) in 0.1 M PBS solution (pH 6.0) containing 20 μM CPZ. The scan rate in (**a**) is 100 mV/s.

In order to examine the analytical performance of VMSF/ErGO/SPCE towards CPZ, CV and DPV curves of bare SPCE, GO/SPCE, ErGO/SPCE, and VMSF/ErGO/SPCE in 0.1 M PBS solution (pH 6.0) containing 20 μM CPZ were recorded. Electrochemical oxidation of CPZ is shown in Scheme S1. As compared in Figure 3a, modification of GO on the SPCE surface causes decreased anodic peak current and increased anodic peak potential of CPZ, which is attributed to the ill conductivity of GO. When GO was electrochemically reduced to the ErGO, remarkably improved anodic peak current was observed at the ErGO/SPCE, confirming the π-π preconcentration ability and good electrocatalytic capacity of ErGO due to the active defects/edge plane sites. After further growth of VMSF bearing negative charges, as illustrated in Figure 1b, VMSF/ErGO/SPCE exhibits excellent performance towards positively charged CPZ (p*K*_a_ ~9.3) in terms of enhanced anodic peak current and decreased anodic peak potential. In addition, the magnitude of anodic peak current at the VMSF/ErGO/SPCE is ca. 9.5-fold larger than that at the bare SPCE (Figure 3b), which arises from the comprehensive effect of ErGO (electrocatalytic ability and π-π preconcentration effect) and VMSF (electrostatic enrichment effect). In addition, the effect of scan rate on the electrochemical behavior of CPZ was investigated. As shown in Figure S1b, in the range from 80 mV/s to 320 mV/s, anodic peak current is linearly proportional to the scan rate, suggesting the electrochemical reaction of CPZ on the VMSF/ErGO/SPCE is adsorption-controlled. The number of electron transfer in the electrochemical reaction of CPZ was calculated using the following Laviron equation:

$$E_{pa}=E^{0}+\left(\frac{RT}{\alpha nF}\right)ln\frac{RTK_{s}}{\alpha nF}+\left(\frac{RT}{\alpha nF}\right)lnv$$

where *E*^0^ is the standard electrode potential; *α* is the transfer coefficient, which is assumed to be 0.5 in the total irreversible electrochemical reaction process; *n* is the number of electron transfers involved in the rate-determining step; *K_s_* is the standard rate constant; and *R* is the gas constant (*R* = 8.314 J mol^−1^ K^−1^), *T* is the absolute temperature (*T* = 298 K), and *F* is Faraday constant (*F* = 96485 C mol^−1^). According to the slope of the fitting curve of *E*_pa_ vs. *v* shown in Figure S1b, the value of *n* is calculated as 1.8, which is close to 2. Therefore, the oxidation of CPZ on the VMSF/ErGO/SPCE is a 2e^−^ transfer process displayed in Scheme S1. 

### 2.3. Electrochemical Detection of CPZ Using VMSF/ErGO/SPCE

**Figure 4:**
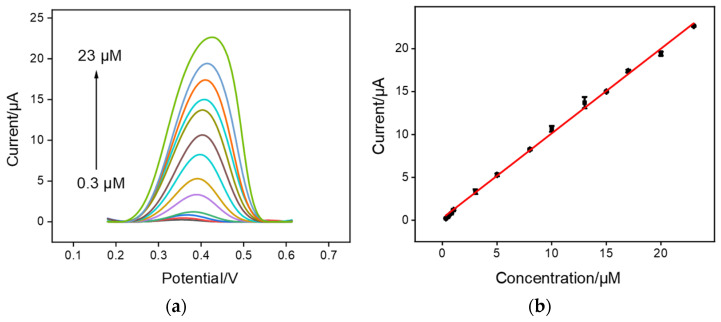
(**a**) DPV curves of VMSF/ErGO/SPCE in 0.1 M PBS solution (pH 6.0) containing various concentrations of CPZ (from bottom to up: 0.3, 0.5, 0.8, 1, 3, 5, 8, 10, 13, 15, 17, 20, and 23 μM). (**b**) The corresponding calibration curve and the error bars represent the standard deviation (SD) of three measurements.

Experimental conditions, including mechanical stirring time and pH of supporting electrolyte, were optimized to achieve highly sensitive performance towards CPZ. As revealed in Figures S2 and S3, the optimal mechanical stirring time and pH of supporting electrolyte are 80 s and 6.0, respectively. Under optimal conditions, VMSF/ErGO/SPCE was applied for CPZ with a series of concentrations and the results are shown in Figure 4. An obvious enhancement of anodic peak current was observed with the successive addition of CPZ to 0.1 M PBS (Figure 4). In addition, the anodic peak current was a linear response to CPZ concentration, yielding a linear dynamic range (0.3~23 μM) (inset of Figure 4). The corresponding linear regressive equations are *I* (μA) = (0.991 ± 0.005) *C* (μM) + (0.00465 ± 0.04970) (*R*^2^ = 0.998) and the limit of detection (LOD) was calculated as 6.1 nM (S/N = 3). Table 1 compares the analytical performances of our VMSF/ErGO/SPCE sensor for CPZ detection with other previously reported electrochemical sensors. As displayed, the proposed VMSF/ErGO/SPCE has a wider linear range, a higher sensitivity, and a lower LOD.

### 2.4. Anti-Interference, Stability and Reproducibility of VMSF/ErGO/SPCE

**Figure 5:**
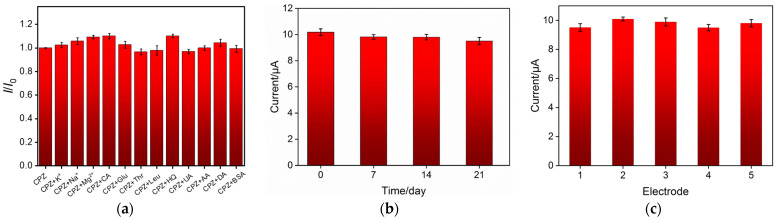
(**a**) The current ratio (*I*/*I*_0_) obtained from VMSF/ErGO/SPCE for detection of 20 μM CPZ in the presence (*I*) and absence (*I*_0_) of added interfering species. The concentration of BSA is 6 μM and the concentration of other interfering species is 1 mM. Stability (**b**) and reproducibility (**c**) of sensors. The error bars represent the SD of three measurements.

Several performance parameters are vital for evaluation of electrochemical sensors, such as anti-interference, stability, and reproducibility. As shown in Figure 5a, interfering substances, including K^+^, Na^+^, Mg^2+^, citric acid (CA), glucose (Glu), threonine (Thr), leucine (Leu), hydroquinone (HQ), ascorbic acid (AA), uric acid (UA), 3-hydroxytyramine hydrochloride (DA), and bovine serum albumin (BSA), were used to examine the anti-interference ability of VMSF/ErGO/SPCE. The presence of interfering substances has not caused significant influence on the detection of CPZ, indicating excellent anti-interference ability. After three-week storage, VMSF/ErGO/SPCE still remains 93.4% of its initial measured value, showing good stability (Figure 5b). Five different VMSF/ErGO/SPCE were prepared using the same procedure and used to detect CPZ, respectively. The obtained relative standard deviation (RSD) from five electrodes is 2.8%, suggesting excellent reproducibility of the proposed sensor (Figure 5c). However, owing to the inherent property of VMSF, the developed VMSF/ErGO/SPCE could not possess long-term stability in strong alkaline solutions.

### 2.5. Real Sample Analysis

**Figure 6:**
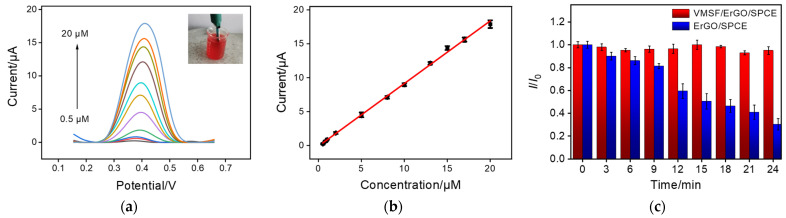
(**a**) DPV curves of VMSF/ErGO/SPCE in human blood samples diluted by a factor of 50 using 0.1 M PBS solution (pH 6.0) containing CPZ ranging from 0.5 μM to 20 μM. Inset is the photograph of human whole blood sample. (**b**) The corresponding calibration curve and the error bars represent the SD of three measurements. (**c**) Normalized anodic peak current ratio (*I*/*I*_0_) at the VMSF/ErGO/SPCE (red) and ErGO/SPCE (blue) towards CPZ (10 μM) in human blood (diluted by a factor of 50). *I* denotes the current obtained in continuous detections at different time and *I*_0_ is the current for the first detection.

Diluted and undiluted human whole blood samples were employed to investigate the practical application of the proposed VMSF/ErGO/SPCE sensor. Figure 6a shows the DPV responses of VMSF/ErGO/SPCE to various concentrations of CPZ in 50-times-diluted human whole blood sample. As presented, in the range from 0.5 μM to 20 μM, anodic peak current was linear with the concentration of CPZ, and the obtained linear regressive equation was *I* (μA) = (0.931 ± 0.010) *C* (μM) – (0.102 ± 0.024) (*R*^2^ = 0.998), with an LOD of 6.5 nM. Note, the sensitivity (0.931 ± 0.010 μA/μM) obtained in 50-times-diluted human whole blood sample is comparable to that obtained in buffer (0.991 ± 0.005 μA/μM). In addition, bare SPCE and ErGO/SPCE were used to detect various concentrations of CPZ using the same procedures. As shown in Figure S4 and Table S1, VMSF/ErGO/SPCE has better analytical performance in terms of wider linear range, higher sensitivity, and lower LOD compared to the bare SPCE and ErGO/SPCE. Long-term stability and regeneration ability of VMSF/ErGO/SPCE in 50-times-diluted human whole blood samples were also studied. It could be found that VMSF/ErGO/SPCE is able to effectively prevent surface fouling of underlying electrode, showing good long-term antifouling and regeneration abilities in 50-times-diluted human whole blood samples (Figure 6a and Figure S5). Figure S6a compares the DPV curves of 3 μM CPZ at the bare SPCE, ErGO/SPCE, and VMSF/ErGO/SPCE in undiluted human whole blood. Obviously, a much sharper anodic peak and higher anodic peak current were observed at the VMSF/ErGO/SPCE in comparison with the ErGO/SPCE and bare SPCE. In addition, the good linear range of 1 μM to 20 μM was obtained at the VMSF/ErGO/SPCE in undiluted human whole blood with a sensitivity of (0.367 ± 0.020) μA/μM and an LOD of 16 nM (Figure S6b). Note that the volume of undiluted human whole blood, as shown in the inset of Figure S6a, is rather small (<50 μL). Moreover, recoveries of CPZ in diluted or undiluted human whole blood samples are within 97.3~104% and RSD is less than 5.1% (Table 2). All these results indicate the developed VMSF/ErGO/SPCE could be applied to detect CPZ in human whole blood with high sensitivity, good stability, good accuracy, and low sample consumption.

## 3. Materials and Methods

### 3.1. Chemicals and Materials

Monolayer graphene oxide (GO) aqueous dispersion (1 mg/g) was purchased from Hangzhou GaoxiTech (Hangzhou, China). Tetraethyl orthosilicate (TEOS), hexadecyl trimethyl ammonium bromide (CTAB), potassium ferricyanide (K_3_[Fe(CN)_6_]), potassium ferrocyanide (K_4_[Fe(CN)_6_]), sodium phosphate dibasic dodecahydrate (Na_2_HPO_4_·12H_2_O), chlorpromazine (CPZ), citric acid (CA), glucose (Glu), *L*-threonine (Thr), *L*-leucine (Leu), hydroquinone (HQ), ascorbic acid (AA), uric acid (UA), 3-hydroxytyramine hydrochloride (DA), and bovine serum albumin (BSA) were obtained from Aladdin Chemistry Co. Ltd. (Shanghai, China). Sodium dihydrogen phosphate dehydrate (Na_2_H_2_PO_4_·2H_2_O) was bought from Macklin (Shanghai, China). Screen-printed carbon electrodes (SPCE) (DRP-C110-U75) were received from Metrohm (Bern, Switzerland). Among them, the working electrode (4 mm in diameter) and counter electrode of the SPCE were prepared from carbon, and Ag was used as a pseudo-reference electrode. Human blood serum (healthy man) was provided from Center for Disease Control and Prevention (Hangzhou, China) for real sample analysis. All reagents used in the experiment were of analytical grade without further treatment. Deionized water (18.2 MΩ cm) was prepared from Mill-Q system (Millipore, Burlington, MA, USA).

### 3.2. Measurements and Instrumentations

X-ray photoelectron spectroscopy (XPS) was performed on PHI5300 electron spectrometer using 250 W, 14 kV, Mg Kα radiation (PE Ltd., Boston, MA, USA). The surface morphology of VMSF was characterized by HT7700 Transmission electron microscope (Hitachi, Tokyo, Japan) at an acceleration voltage of 100 kV. VMSF was prepared by gently scraping from the surface of SPCE and was dispersed in the ethanol. The dispersion was further dropped onto the copper grids after ultrasonication for 2 h to obtain the TEM specimen. All electrochemical experiments, including cyclic voltammetry (CV), differential pulse voltammetry (DPV), and electrochemical impedance spectroscopy (EIS), were carried out on an Autolab (PGSTAT302N, Metrohm) electrochemical workstation. In this article, the scan rate used in CV was 100 mV/s, and DPV parameters used were as follows: step, 0.005 V; modulation amplitude, 0.025 V; modulation time, 0.05 s; interval time, 0.2 s.

### 3.3. Preparation of VMSF/ErGO/SPCE

Prior to use, SPCE first underwent electrochemical pretreatment to remove the impurities absorbed on the electrode surface by applying a potential range of 0.4–1.0 V in 0.05 M H_2_SO_4_ for 10 cycles. After being washed by ultrapure water and dried under nitrogen, 20 μL of GO (0.1 mg/mL) was dropped onto the SPCE and dried at 60 °C. The electrode was named as GO/SPCE. VMSF was grown onto the GO/SPCE surface according to the protocol reported previously [55]. Briefly, the precursor solution was prepared by mixing 40 mL 0.1 M NaNO_3_ and EtOH (*v*/*v* = 1:1), 1.585 g CTAB, and 3050 μL TEOS under vigorous stirring for 2.5 h. Subsequently, GO/SPCE was placed into the above precursor solution and subjected to a constant voltage of –2.1 V for 5 s. Then, the electrode was quickly washed by deionized water, dried by nitrogen, and further aged at 80 °C. Thus, the resulting electrode with surfactant micelles (SM) inside the nanochannels was designed as SM@VMSF/ErGO/SPCE. In order to remove SM from the silica nanochannels, SM@VMSF/ErGO/SPCE was put into 0.1 M HCl-EtOH under stirring for 5 min. The obtained electrode was called VMSF/ErGO/SPCE.

### 3.4. Real Sample Analysis

The received human whole blood was diluted or undiluted with 0.1 M PBS (pH 6.0) without any complex pretreatment. Upon the addition of known concentration of CPZ into the human whole blood samples, electrochemical responses of CPZ were determined by the VMSF/ErGO/SPCE sensor. The volume of samples is very small (<50 µL).

## 4. Conclusions

In summary, we present a simple and efficient method for preparation of disposable VMSF/ErGO/SPCE electrochemical sensor and investigate its analytical performance of CPZ detection. Such VMSF/ErGO/SPCE could be obtained by a rapid and controllable one-step electrochemical method, which combines the reduction of GO and growth of VMSF. Arising from the electrostatic preconcentration ability of outer VMSF layer and electrocatalytic capacity and π-π enrichment ability of inner ErGO layer, the proposed VMSF/ErGO/SPCE shows excellent analytical performance towards CPZ. Furthermore, combining the inherent characteristics of VMSF (antifouling ability) and SPCE (disposable property), direct electrochemical analysis of CPZ in human whole blood was successfully realized with rather low sample consumption and good anti-interference, stability, reproducibility, and regeneration abilities, which could be extended to detect various analytes related to diseases.

## Figures and Tables

**Table 1:** Comparison of the analytical performances of different electrochemical sensors for the determination of CPZ.

Electrode	Technique	Linear Range (μM)	Electrolyte	LOD (nM)	Ref.
N-CDs/Cu_2_O/Nf/GCE	DPV	0.001–230	PBS	25	[13]
RGO@PDA/GCE	Amperometry	0.03–967.6	PBS	1.8	[52]
SPO/PPy/SPCE	DPV	0.8–1207 5–30	PBS human serum	69 /	[53]
SrM/SPCE	DPV	0.1–1683	PBS	28	[2]
RuS_2_/AG/SPCE	DPV	0.05–1249	PBS	8	[54]
VMSF/ErGO/SPCE	DPV	0.3–23 1–10	PBS whole blood	6.1 16	Our work

N-CDs/Cu_2_O/Nf/GCE: Nafion (Nf)-supported nitrogen-doped carbon dots/cuprous oxide composite. RGO@PDA/GCE: graphene oxide (RGO) and polydopamine (PDA) composite GCE. SPO/PPy/SPCE: alpha-phase strontium pyrophosphate incorporated polypyrrole-modified SPCE. SrM/SPCE: Strontium-molybdate-modified SPCE. RuS_2_/AG/SPCE: Ruthenium sulfide nanoparticles-decorated activated graphite sheets modified SPCE.

**Table 2:** Recoveries of CPZ in diluted or undiluted human whole blood samples.

Sample	Added (μM)	Found (μM)	RSD (%)	Recovery (%)
diluted human whole blood ^a^	1.00	1.03	2.1	103
	5.00	4.98	3.3	99.6
	10.0	9.66	2.2	96.6
	15.0	15.6	1.8	104
undiluted human whole blood	3.00	2.98	5.1	99.3
	5.00	4.87	3.0	97.4
	10.0	9.73	3.4	97.3

^a^ Human blood samples were diluted by a factor of 50 using PBS (0.1 M, pH 6.0) buffer solution before a series of CPZ were added.

## Data Availability

The data presented in this study are available on request from the corresponding author.

## Supplementary Materials

The following supporting information can be downloaded at: https://www.mdpi.com/article/10.3390/molecules27238200/s1, Scheme S1: electrochemical reaction of CPZ on the electrode surface; Figure S1: effect of scan rate on the CV responses; Figure S2: effect of preconcentration time on the detection performance; Figure S3: effect of pH of supporting electrolyte on the detection performance; Figure S4: electrochemical detection of CPZ in diluted human whole blood using bare SPCE and ErGO/SPCE; Table S1: comparison of electrochemical detection performance towards CPZ at the various electrodes in human whole blood samples; Figure S5: anti-interference, stability and reproducibility ability; Figure S6: regeneration ability; Figure S7: electrochemical detection of CPZ in undiluted human whole blood samples.
