# Supplementary material for: Disposable Electrochemical Sensors for Highly Sensitive Detection of Chlorpromazine in Human Whole Blood Based on the Silica Nanochannel Array Modified Screen-Printed Carbon Electrode
Source: Molecules. 2022 Nov 24;27(23):8200. doi: 10.3390/molecules27238200 (PMC9735719; doi:10.3390/molecules27238200)
Supplement: Supplementary file 1 [file molecules-27-08200-s001.zip › molecules-2002009-supplementary.pdf]

Supporting Information to

Disposable electrochemical sensors for highly sensitive detection of chlorpromazine in human whole blood based on the silica nanochannel array modified screen-printed carbon electrode

Qianqian Han[1,‡], Tongtong Zhang[2,‡], Meifang Wang[1], Fei Yan[1,] and Jiyang Liu[1,*]*

[1] Key Laboratory of Surface & Interface Science of Polymer Materials of Zhejiang Province, Department of Chemistry, Zhejiang Sci-Tech University, Hangzhou 310018, China

[2] Key Laboratory of Integrated Oncology and Intelligent Medicine of Zhejiang Province, Department of Hepatobiliary and Pancreatic Surgery, Affiliated Hangzhou First People's Hospital, Zhejiang University School of Medicine, Hangzhou, 310006, China

‡ These two authors contributed equally.

* Corresponding author. E-mail: feifei19881203@126.com or yanfei@zstu.edu.cn (F.Y.); liujy@zstu.edu.cn (J.L.)

Table of Contents

S1. Electrochemical reaction of CPZ on the electrode surface

S2. Effect of scan rate on the CV responses

S3. Optimization of experimental conditions

S3.1 preconcentration time

S3.2 pH of supporting electrolyte

S4. Electrochemical detection of CPZ in human whole blood using bare SPCE and ErGO/SPCE

S5. Anti-interference, stability and reproducibility ability of VMSF/ErGO/GCE

S6. Regeneration ability of VMSF/ErGO/GCE, ErGO/SPCE and bare SPCE

S7. Electrochemical detection of CPZ in undiluted human whole blood samples

S1. Electrochemical reaction of CPZ on the electrode surface

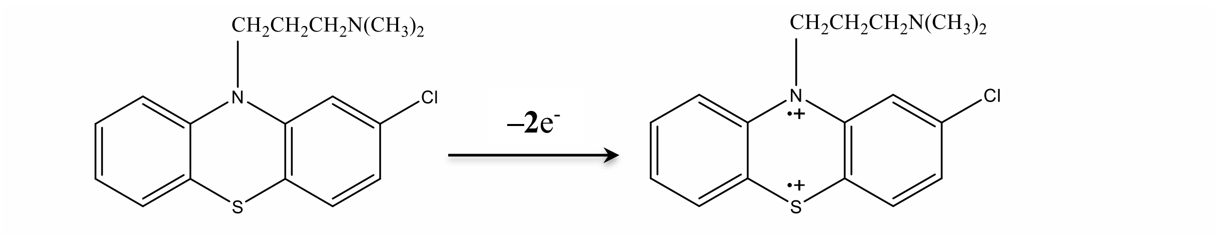

Scheme S1 Electrochemical reaction of CPZ on the electrode surface.

S2. Effect of scan rate on the CV responses

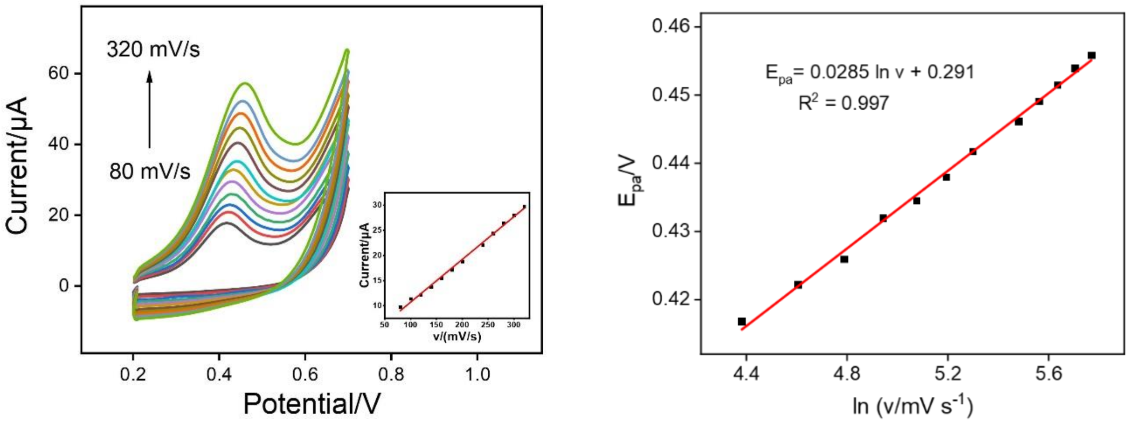

(**a**) (**b**)

Figure S1 (**a**) Effect of scan rates (*v*) on the redox behavior of 20 μM CPZ in 0.1 M PBS (pH 6.0) at the VMSF/ErGO/SPCE. Inset depicts the linear relationship of anodic peak current with scan rate. (**b**) Relationship between anodic peak potential (E_{pa}) and ln *v*.

S3. Optimization of experimental conditions

S3.1 preconcentration time

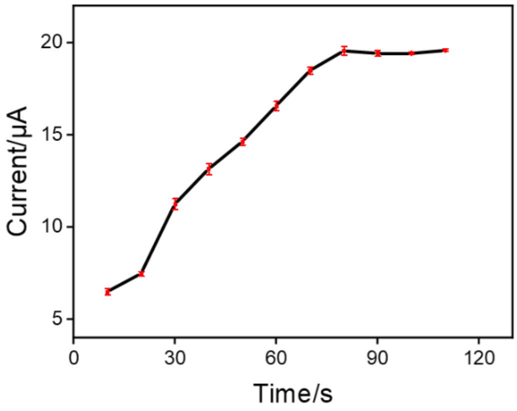

Figure S2 Time-dependent plots at the VMSF/ErGO/SPCE in 0.1 M PBS solution (pH 6.0) containing 20 μM CPZ. The error bars represent the standard deviation (SD) of three measurements.

S3.2 pH of supporting electrolyte

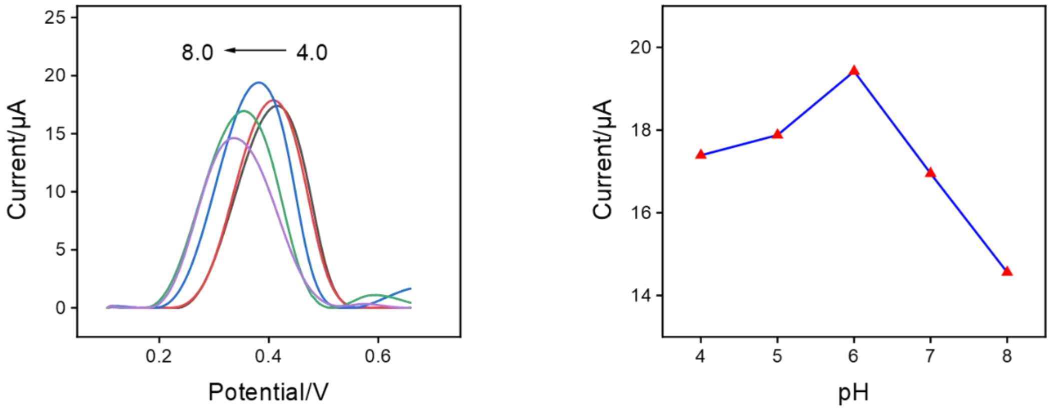

(**a**) (**b**)

Figure S3 (**a**) DPV curves obtained at the VMSF/ErGO/SPCE in 0.1 M PBS solution containing 20 μM CPZ at various pH values. (**b**) Effect of pH of supporting electrolyte on the anodic peak current of CPZ.

S4. Electrochemical detection of CPZ in human whole blood using bare SPCE and ErGO/SPCE

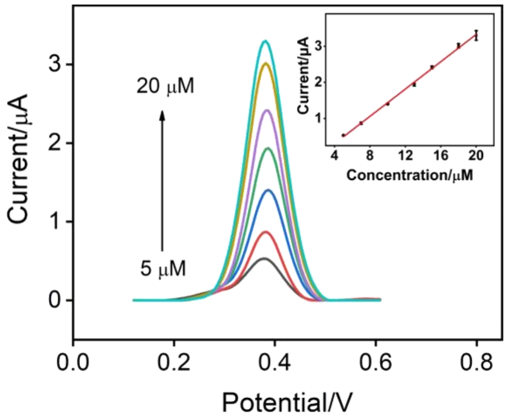

(**a**)

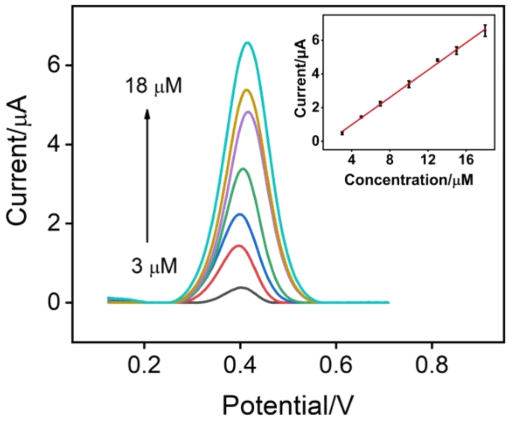

(**b**)

Figure S4 DPV curves of bare SPCE (**a**) and ErGO/SPCE **(b**) in human blood samples diluted by a factor of 50 using 0.1 M PBS solution (pH 6.0) containing different concentrations of CPZ. Insets are corresponding calibration curves and the error bars represent the SD of three measurements.

Table S1. Comparison of electrochemical detection performance towards CPZ at the various electrodes in 50-time diluted human whole blood samples

Electrode	Linear range (μM)	Sensitivity (μA μM^{-1})	LOD (nM)
bare SPCE	5–20	0.1889	31.8
ErGO/SOCE	3–18	0.4050	14.8
VMSF/ErGO/SPCE	0.5–20	0.9239	6.5

S5. Anti-interference, stability and reproducibility ability of VMSF/ErGO/GCE

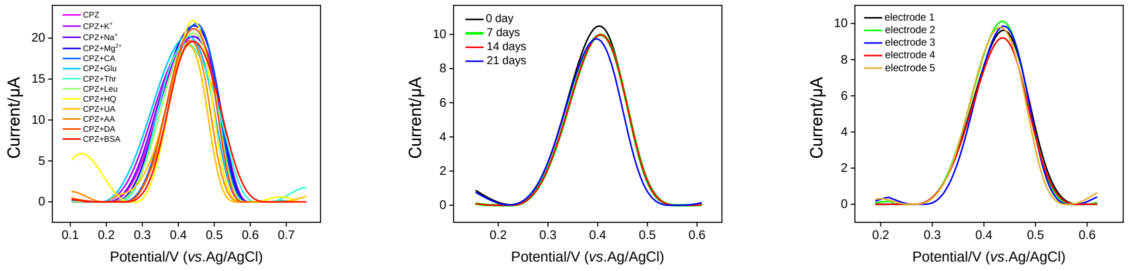

(**a**) (**b**) (**c**)

Figure S5 (**a**) DPV curves obtained from VMSF/ErGO/SPCE for detection of 20 μM CPZ in the presence and absence of added interfering species. The concentration of BSA is 6 μM and the concentration of other interfering species are 1 mM. Stability (**b**) and reproducibility (**c**) of sensors.

S6. Regeneration ability of VMSF/ErGO/GCE, ErGO/SPCE and bare SPCE

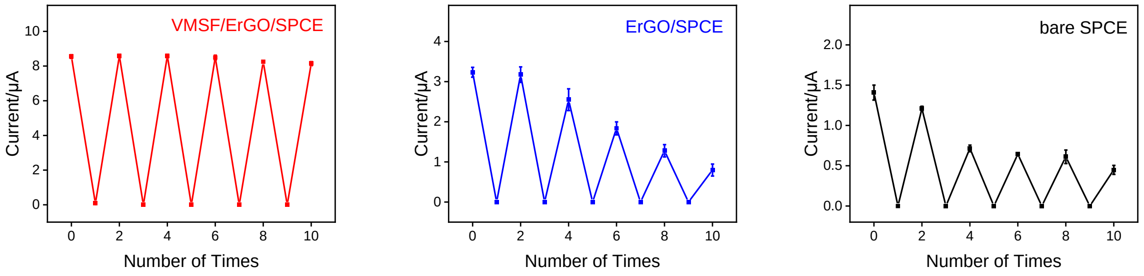

(**a**) (**b**) (**c**)

Figure S6 Peak currents and usage times curves of VMSF/ErGO/SPCE (**a**), ErGO/SPCE (**b**) and bare SPCE (**c**) in 50-time diluted human blood samples with or without 10 μM CPZ. To realize the regeneration, electrodes were soaked into a HCl-ethanol solution.

S7. Electrochemical detection of CPZ in undiluted human whole blood samples

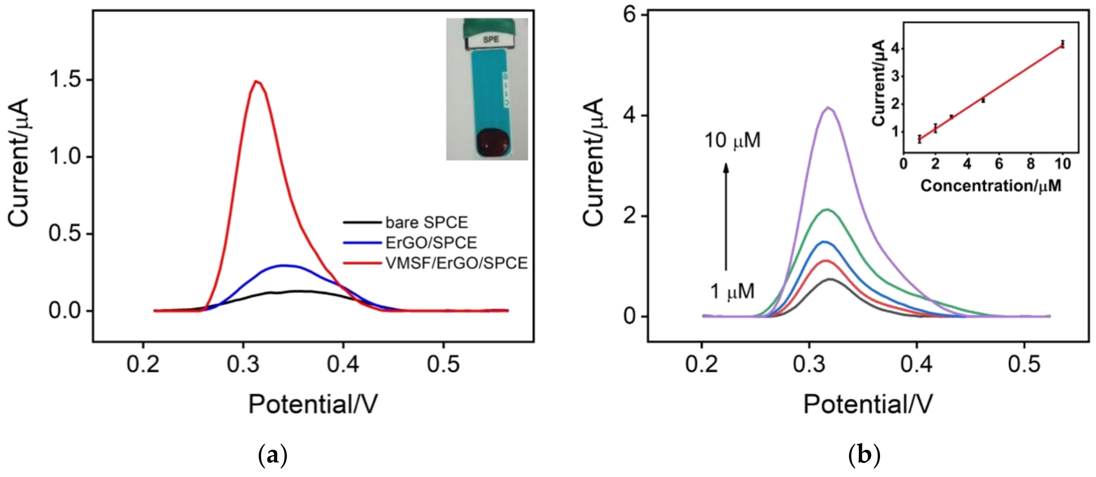

Figure S7 (**a**) DPV curves of bare SPCE, ErGO/SPCE and VMSF/ErGO/SPCE in human whole blood samples containing 3 μM CPZ; Inset is the photograph of VMSF/ErGO/SPCE used for analysis of CPZ in undiluted human whole blood. (**b**) DPV curves of VMSF/ErGO/SPCE in human blood samples containing CPZ ranging from 1 μM to 10 μM. Inset is the corresponding calibration curve and the error bars represent the SD of three measurements.
